# 1838. Determinants of Antimicrobial Resistance among ≤5-Year Children in Low-Income Urban Bangladesh

**DOI:** 10.1093/ofid/ofad500.1667

**Published:** 2023-11-27

**Authors:** Md Golam Dostogir Harun, Shariful Amin Sumon, Syed Abul Hassan Md Abdullah, Md Saiful Islam, Mahabub Ul Anwar

**Affiliations:** icddrb, Dhaka, Dhaka, Bangladesh; icddr,b, Mohakhali, Dhaka, Bangladesh; SafetyNet Bangladesh, Dhaka, Dhaka, Bangladesh; UNSW Sydney NSW, Sydney, New South Wales, Australia; West Virginia University, Morganton, West Virginia

## Abstract

**Background:**

Antimicrobial resistance (AMR) is a growing global concern, with the most significant health and financial implications for low- and middle-income countries. Due to irrational and inappropriate use, AMR is rapidly increasing in Bangladesh, especially among low-income urban populations. This study explored the factors for antimicrobial resistance among ≤ 5 years children in low-income urban Bangladesh.

**Methods:**

From February 2018 to April 2019, we did quantitative and qualitative assessments of antibiotic use among urban ≤5-year children. We conducted a cross-sectional survey among 626 low-income urban ≤5-year children's parents in four cities in Bangladesh. We used a semi-structured questionnaire to collect data related to antibiotic use through face-to-face interviews. We also conducted eight focus group discussions among the parents to gather in-depth information on antibiotic use to treat their children. Bivariate and multivariate analysis was done to see the causes and associated factors.

**Results:**

The study identified that 62.9 % (394/626) of parents used antibiotics last year to treat their children. More than half (52.7%) of the parents bought antibiotics from local drug stores without a prescription. 46.5% (291/626) of the parents stopped antibiotics soon after the symptom disappeared, and 62.6% used leftover antibiotics for similar symptoms. However, 82.9% of parents opined that qualified physicians' advice is important to treat children with antibiotics. Inappropriate antibiotic use was significantly associated with participants' education (p=0.02), family income (p=0.01), and profession (p=0.04). Qualitative investigation revealed the convenience of getting treatment and medicine from the drug store, the financial burden to buy the full course, the distance and waiting time at qualified physicians, and parents' inadequate knowledge were identified as the key causes of irrational antibiotic use.

Pattern of Antibiotic Consumption among ≤5-Year Children in Low-Income Urban Bangladesh

**Figure:**
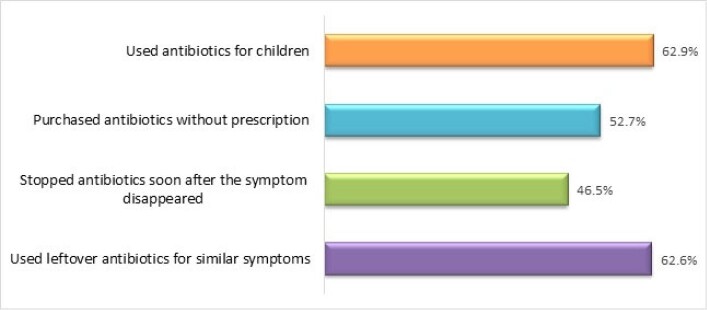

**Conclusion:**

Intervention packages are required to improve parents’ knowledge and awareness on the rational use of antibiotic. Strengthening the health system could enhance the optimum use of antibiotics and combat antibiotic resistance. Antibiotic dispensing, prescription, purchase, and selling policy required with enforcement of the policy to reduce AMR.

**Disclosures:**

**All Authors**: No reported disclosures

